# Association between dietary inflammatory index and components of metabolic syndrome

**DOI:** 10.34172/jcvtr.2020.05

**Published:** 2019-12-24

**Authors:** Sima Ghorabi, Alireza Esteghamati, Kamal Azam, Elnaz Daneshzad, Omid Sadeghi, Asma Salari-Moghaddam, Leila Azadbakht, Kurosh Djafarian

**Affiliations:** ^1^Department of Clinical Nutrition, School of Nutritional Sciences and Dietetics, Tehran University of Medical Sciences, Tehran, Iran; ^2^Endocrinology and Metabolism Research Center (EMRC), Vali-Asr Hospital, Tehran University of Medical Sciences, Tehran, Iran; ^3^Department of Epidemiology and Biostatistics, School of Public Health, Tehran University of Medical Sciences, Tehran, Iran; ^4^Department of Community Nutrition, School of Nutritional Sciences and Dietetics, Tehran University of Medical Sciences, Tehran, Iran

**Keywords:** Metabolic Syndrome, Dietary Inflammatory Index, Diet, Inflammation

## Abstract

*** Introduction:*** Limited data are available on the association of Dietary Inflammatory Index (DII) with metabolic syndrome (MetS) and its components. The present study was conducted to investigate the association of DII with MetS and its components among Iranian adults.

***Methods: *** A total of 404 subjects, aged 18 years or older, were included in the current cross-sectional study. We used a validated and reliable 147-item food frequency questionnaire (FFQ) to assess dietary intakes. Fasting blood sample was obtained to quantify glycemic indicators and lipid profile. MetS was defined based on the guidelines of the National Cholesterol Education Program Adult Treatment Panel III (ATP III).

*** Results:*** Mean age of study participants was 38.20 ± 9.55 years. No significant association was found between DII and odds of MetS (odds ratio [OR]: 0.92, 95% CI: 0.48-1.76). In terms of MetS components, a significant positive association was seen between DII scores and reduced levels of high-density lipoprotein cholesterol (HDL-C) (OR: 2.29, 95% CI: 1.32-3.97); such that after controlling for energy intake, demographic variables and BMI, participants in the highest category of DII had 2.71 times greater odds for having reduced levels of HDL-C (OR: 2.71, 95% CIs: 1.34, 5.47). There was no other significant association between other components of MetS and DII scores either before or after adjusting for confounding variables.

*** Conclusion:*** We observed no significant association between DII and odds of MetS. However, higher score of DII was associated with lower levels of HDL.

## Introduction

Metabolic syndrome (MetS) refers to a set of metabolic disorders including lipid disorders, deviant glucose homeostasis, abdominal obesity, and high blood pressure. All mentioned metabolic disorders are associated with an increased risk of diabetes mellitus, non-alcoholic fatty liver disease, chronic kidney disease, cardiovascular diseases (CVDs), stroke, some cancers and even mortality.^1^ MetS is increasing throughout the world in both developing and developed countries. It has been estimated that 25%^2^ of general population are affected. In Iran, this syndrome is prevalent in 23.8% of adults 20 years and older.^3^

The etiology of MetS is not well known, however, it has been shown that a combination of genetic and environmental factors contribute to this syndrome.^4,5^ Among environmental factors, diet has an important role in MetS development.^6^ Previous studies have revealed that dietary intake of red meat, cholesterol, saturated and trans-fatty acids and iron-containing foods is positively associated with the risk of MetS.^7-11^ These food items increase the inflammatory potential of diet. It means that consumption of these foods can increase the inflammatory biomarkers in blood. On the other hand, inflammation is a well-known risk factor of MetS. Therefore, there might be a link between the dietary inflammation capacity and MetS.^12-14^ However, previous studies have mainly assessed the intake of a single food with high inflammatory potential in relation to MetS and little attention has been laid on considering diet as whole. Assessing total inflammatory potential of diet in evaluation of diet-disease relations is better than assessing the intake of a single food or nutrient. Focusing on a whole diet is better due to decreasing the co-linearity problem which might occur when investigating single nutrient and food intakes.^15^

Few studies have investigated the association between Dietary Inflammatory Index (DII) and MetS and their findings are conflicting. A prospective study in France and cross-sectional study in Iran showed that higher DII was associated with higher risk of MetS,^16,17^ while two studies did not find any significant association.^18,19^ Previous investigations on the association between DII and MetS have been confined to western nations and limited documents are available from the understudied region of the Middle East, where the prevalence of MetS is seemed to be high. Therefore, the present study aimed to examine the association between inflammatory potential of diet and MetS in Iranian adults.

## Materials and Methods

### Participants

In the current study, participants were recruited from the Endocrine Clinic of Imam Khomeini hospital, Tehran University of Medical Sciences (TUMS), Tehran, Iran. Written informed consent was signed by each participant before data gathering. Based on the previous study^20^ which 42% of the population had unhealthy dietary pattern and considering 95% confidence interval, sample size was calculated about 375 individuals ((Zl-α/2×P(1-P))/d^2^= (1.96^2^×0.42)(1-0.42)/(0.05)^2^= 375). However, according to some exclusion criteria and to prevent data reduction, we continued sampling. Subjects were selected by convenience sampling method based on inclusion and exclusion criteria. This study was conducted between April 2017 and March 2018. Inclusion criteria were age of ≥18 years and tendency to participate in this study. In contrast, we excluded individuals who were professional athlete, in pregnancy, menopause and lactation period, those who suffered from any type of cancer, liver or kidney diseases, individuals who used medication for modifying lipid profile, blood sugar, hypertension, and for treatment of hyper- or hypothyroidism and ischemic heart disease. In addition, use of sedative or hypnotic drugs, antihistamine and immune system inhibitors were other exclusion criteria. Furthermore, individuals who adhered to a special diet or used dietary supplements were also excluded. Finally, 404 individuals were included in the analysis.

### Dietary intakes

A validated 147-item food frequency questionnaire (FFQ) was used to assess dietary intakes. Participants were instructed how to fulfill the questionnaire by one trained dietitian. FFQ presents a list of food items and a standard serving size for each one. Participants should report the frequency of their foods consumption during the previous year (frequency of food items on daily, weekly, or monthly intake). The portion size of consumed foods was converted to grams using booklet of “household measures”. Daily nutrients intake for each subject was calculated using the US Department of Agriculture’s (USDA) national nutrient databank.^21^ The FFQ provided reliable and validated data on long-term intake of foods and nutrients.^22^

### Development of DII

We used the method of Shivappa et al to determine DII scores.^23^ Construction of this method was validated by previous studies.^24,25^ Shivappa et al reported a total of 45 specific nutrients and foods affecting the concentrations of some inflammatory biomarkers such as interleukin- 6 (IL-6), interleukin-1b (IL-1b), tumor necrosis factor-a (TNF-a) and C-reactive protein (CRP) or anti-inflammatory biomarkers such as interleukin-10 (IL-10) and interleukin-4 (IL-4). Then, the inflammatory potential of each food item was scored according to whether it increased inflammatory or decreased anti-inflammatory factors (+1), or it decreased inflammatory or increased anti-inflammatory factors (-1) or had no effect (0) on inflammatory or anti-inflammatory biomarkers. In Shivappa et al method, world mean and standard deviation for each of the 45 food items were calculated according to eleven database from 11 countries in several parts of the world. In this study, DII score was calculated according to 30 food parameters (rather than 45), due to lack of consumption of some foods in Iranian dietary culture as well as missing some items (like polyphenols) in Iranian nutrient database. We had 8 pro-inflammatory parameters including energy, protein, total fat, carbohydrates, saturated fatty acids, trans fat, iron, and cholesterol and 22 anti-inflammatory parameters including polyunsaturated fatty acids (PUFA), monounsaturated fatty acids (MUFA), fiber, magnesium, selenium, zinc, β-carotene, folic acid, caffeine, vitamins B1, B2, B3, B6, B12, C, E, A and D, garlic, onion, tea, and pepper. First, energy-adjusted amounts of these items were calculated using residual method. Then, the z-score was calculated for each participant by subtracting the “standard global mean” from the amount consumed by each subject and dividing this value by the “global standard deviation”. Both “standard global mean” and “global standard deviation” were derived from study of Shivappa et al. The obtained z-score was then converted to a centered percentile score in order to reduce skewness, as earlier studies did.^23^ For each participant, this score was multiplied by the respective food parameter effect score derived from the study of Shivappa et al.^23^ Then, overall DII score for each participant was calculated by summing up DII score obtained for all 30 food parameters. Higher DII score (more positive) presents a more inflammatory diet and lower DII score (more negative) presents a less inflammatory diet.

### Anthropometric measures

Height was measured in a standing position without wearing shoes, using a measuring tape while shoulders were relaxed. Weight was measured while the subjects were light clothed and without wearing shoes using a Seca (Hanover, MD) portable scale. Waist circumference (WC) was measured using a flexible anthropometric tape midway between the iliac crest and lower rib margin. Body mass index (BMI) was calculated by dividing weight to height (kg/m^2^).

### Biochemical assessments

Blood sample was collected after 12 h overnight fasting to quantify serum levels of fasting blood sugar (FBS), triglyceride (TG), and high-density lipoprotein (HDL). FBS was measured by enzymatic colorimetric method using glucose oxidase. Serum TG concentrations were assayed using enzymatic colorimetric tests with glycerol phosphate. HDL was measured after precipitation of the apolipoprotein B containing lipoproteins with phosphotungstic acid.

### Assessment of other variables

Blood pressure was measured taken from the right arm in a seated and relaxed position by using a standard mercury sphygmomanometerand after subjects rested for 10 minutes. Blood pressure was measured 3 times with a 5-minute interval and finally, mean of these 3 measurements was recorded as the blood pressure variable. Furthermore, a self-administered questionnaire was used to gather data on age, gender (male/female), marital status (single/married), economic status (weak/good), education (under university/ university graduated), smoking (non-smoker/former smoker/current smoker), medications (any types of drugs) and intake of nutritional supplements (minerals, vitamins, iron and calcium). Physical activity (PA) was assessed using the international physical activity questionnaire (IPAQ). Participants were classified into two categories based on PA: inactive and physically active. To determine economic status, we considered income of participants; having ≥2 million tomans income per month as good and <2 million tomans per month as weak economic status.

### Definition of term

MetS was defined according to National Cholesterol Education Program (NCEP ATP III) criteria.^26^ Presence of 3 or more of the following criteria was considered as MetS: 1) abdominal obesity [WC ≥88 cm for women and ≥102 cm for men]; 2) high serum TG [≥150 mg/dL]; 3) low HDL concentrations [<50 mg/dL for women and <40 mg/dL for men]; 4) abnormal glucose homeostasis [FBS >100 mg/dL]; and 5) elevated blood pressure [systolic blood pressure ≥130 mmHg and/or diastolic blood pressure ≥85 mm Hg].

### Statistical analysis

The chi-square test was applied to assess the distribution of categorical variables across tertiles of DII. One-way ANOVA test was used to determine differences in continuous variables and to compare dietary intakes of participants across tertiles of DII scores. Binary logistic regression was used to determine the association between DII and MetS in crude and adjusted models. In the first model, we controlled age, gender, and energy intake. Further adjustment was made for marital status (single/married), physical activity (continuous), education (under university/ university graduated), smoking (non-smoker/ current smoker), economic status (weak/good) and intake of nutritional supplements in the second model. In the final model, BMI was controlled to reach general obesity-independent association between DII and MetS. In all models, participants in the first tertile of DII were considered as the reference group. To obtain the overall trend of odds ratios across increasing tertiles of DII, we considered these tertiles as an ordinal variable in the logistic regression models. In addition to MetS, we also performed the same analyses for components of this syndrome in relation to DII. All statistical analyses were conducted using the Statistical Package for Social Sciences (version 20; SPSS Inc.). *P* < 0.05 was considered as statistically significant.

## Results

The mean age of participants was 38.20±9.55 years and 37.6% were female. Prevalent of MetS was 36.6% (148).

Demographic characteristics, biochemical parameters and anthropometric measures of study participants across tertiles of DII and based on having and not-having MetS are indicated in Table 1. Compared with subjects in the first tertile of DII, those in the third tertile were younger and had lower levels of physical activity. In addition, the prevalence of abnormal glucose homeostasis was significantly different among tertiles of DII. Participants in the top tertile of DII had higher prevalence of reduced serum HDL-C compared with subjects in the lowest tertile. No other significant difference was observed in terms of other anthropometric measures, demographic variables, and biochemical parameters among tertiles of DII.

**Table 1:** Baseline characteristics of study participants across tertiles of DII scores (n = 404)

**Variables**	**Tertilesof Dietary Inflammatory Index**			***P*** **value** ^*^
	**T1**	**T2**	**T3**	
n	134	135	135	
Age (y)	39 ± 9.83	39.28 ± 10.10	36.31 ± 8.41	0.018
Gender (female) (%)	31.3	38.1	30.6	0.300
Weight (kg)	80.35 ± 15.97	81.91 ± 14.91	84.55 ± 19.64	0.123
BMI (kg/m^2^)	28.57 ± 4.99	29.02 ± 4.75	29.01 ± 5.85	0.725
WC (cm)	96.06 ± 12.04	98.01 ± 12.77	98.88 ± 13.81	0.187
Marital status (married) (%)	35.3	33.7	31	0.208
Current smoker,%	18	23	29	0.217
Education(university graduated),%	30.7	34.9	34.3	0.666
Physical activity	1792.4± 2988.5	1429.3± 2426	1044.1± 1373	0.042
Economic status (good)	36.3	31.5	32.2	0.053
SBP (mm Hg)	79.91 ± 49.51	72.45 ± 51.94	68.09 ± 52.12	0.161
DBP (mm Hg)	53.73 ± 33.14	48.56 ± 34.26	45.77 ± 35.04	0.154
FBS (mg/dL)	99.59 ± 20.62	105.25 ± 31.17	99.20 ± 25.56	0.106
TG (mg/dL)	155.87± 131.91	157.75± 122.60	152.20± 125.62	0.935
HDL (mg/dL)	51.86 ± 8.56	51.25 ± 9.76	49.58 ± 10.61	0.137
Component of MetS				
Abdominal adiposity	29.9	35	35	0.434
Elevated blood pressure	38.6	31.8	29.7	0.012
High serum triacylglycerol	34.4	33.6	32	0.904
Reduced serum HDL-C	29.9	29.9	40.2	<0.0001
Abnormal glucose homeostasis	29.3	42	28.7	0.016

Data are presented as mean (SD) or percent. Abbreviations: BMI, body mass index; WC, waist circumference; FBS, fasting blood glucose; TG, triglyceride; HDL: high-density lipoprotein; SBP, systolic blood pressure; DBP, diastolic blood pressure. ^*^ *P* values are resulted from analysis of one-way ANOVA and chi-square test, where appropriate.

Dietary intakes of study participants among tertiles of DII scores are presented in Table 2. Participants in the highest tertile of DII score had higher intakes of energy and total cholesterol and lower intakes of carbohydrate, total fat, PUFA, protein, fiber, magnesium, zinc, iron, folate, selenium, vitamin A, D, E, B6, and C, thiamin, riboflavin, niacin and tea compared with those in the lowest tertile. No other significant association was seen in this regard.

**Table 2:** Dietary intakes of study participants across tertiles of DII score and MetS status (n = 404)

**Variables**	**Tertilesof Dietary Inflammatory Index**			***P*** **value** ^*^
	**T1**	**T2**	**T3**	
Energy (kcal/d)	1739.6 ± 3881	2881 ± 1844.1	3056.9 ± 1483.4	<0.0001
Carbohydrate (g/d)	426.02 ± 6.80	415.80 ± 6.59	378.69 ± 6.66	<0.0001
Protein (gr/d)	215.49 ± 7.67	154.26 ± 7.43	110.82 ± 7.51	<0.0001
Total fat (g/d)	181.06 ± 8.6	150.36 ± 8.33	114.31 ± 8.42	<0.0001
Cholesterol (mg/d)	219.59 ± 12.30	225.28 ± 11.92	269.10 ± 12.05	0.007
Fiber (g/d)	71.12 ± 1.63	57.61 ± 1.58	41.01 ± 1.60	<0.0001
PUFA (g/d)	25.82 ± 0.94	23.56 ± 0.92	20.00 ± 0.93	<0.0001
MUFA (g/d)	27.75 ± 0.88	27.93 ± 0.85	27.26 ± 0.86	0.848
SFA (g/d)	31.61 ± 1.21	29.03 ± 1.17	30.48 ± 1.18	0.318
Iron (mg/d)	59.83 ± 2.02	41.76 ± 1.96	15.90 ± 1.98	<0.0001
Magnesium (mg/d)	725.72 ± 15.57	589.75 ± 15.08	467.00 ± 15.25	<0.0001
Zinc (mg/d)	18.81 ± 0.44	15.67 ± 0.43	14.05 ± 0.44	<0.0001
Folate (mcg/d)	834.42 ± 14.5	728.04 ± 14.05	624.7 ± 14.21	<0.0001
Selenium (mcg/d)	174.04 ± 7.99	168.27 ± 7.74	145.47 ± 7.83	0.028
Vitamin A (RE)	2376.3 ± 107.98	957.8 ± 104.61	700.25 ± 105.76	<0.0001
Vitamin D (mcg/d)	2.97 ± 0.13	2.17 ± 0.12	1.78 ± 0.12	<0.0001
Vitamin C (mg/d)	380.81 ± 18.87	248.54 ± 18.87	144.39 ± 18.48	<0.0001
Vitamin E (mg/d)	19.71 ± 0.49	17.16 ± 0.48	13.38 ± 0.48	<0.0001
Thiamin (mg/d)	2.8 ± 0.087	2.64 ± 0.084	2.36 ± 0.085	0.002
Riboflavin (mg/d)	3.22 ± 0.059	2.55 ± 0.057	2.04 ± 0.057	<0.0001
Niacin (mg/d)	35.6 ± 0.83	30.48 ± 0.809	26.01 ± 0.81	<0.0001
Vitamin B6 (mg/d)	3.26 ± 0.05	2.51 ± 0.04	1.99 ± 0.05	<0.0001
Vitamin B_12_ (mcg/d)	5.67 ± 0.38	5.09 ± 0.37	4.79 ± 0.37	0.633
Caffeine (g/d)	146.39 ± 8.70	168.35 ± 8.43	140.93 ± 8.52	<0.0001
Garlic (mcg/d)	14.66 ± 1.50	15.21 ± 1.51	13.26 ± 1.49	0.065
Onion (g/d)	31.19 ± 3.15	22.75 ± 3.15	12.44 ± 3.14	0.186
Green/Black tea (g/d)	1.37 ± 0.065	1.17 ± 0.06	1.15 ± 0.64	0.034
Pepper (g/d)	14.05 ± 0.63	14.77 ± 0.61	15.58 ± 0.62	0.241

All values are presented as mean (SD). *Obtained by multivariate analysis of variance.

Multivariable-adjusted odds ratios (ORs) and 95% CIs for MetS and its components among tertiles of DII scores are presented in Table 3. No significant association was found between DII score and odds of MetS (OR: 0.92, 95% CI: 0.48, 1.76). Such finding was also observed even after controlling for confounders. However, a significant positive association was found between DII scores and reduced levels of HDL-C; such that after adjustment for demographic variables, energy intake, and BMI, subjects in the highest category of DII had 2.71 times greater odds for having reduced levels of HDL-C (OR: 2.71, 95% CIs: 1.34, 5.47). There was no other significant association between other components of MetS and DII scores either before or after adjusting for confounding variables.

**Table 3:** Odd ratios (95% CI) for MetS across tertiles of DII

**Variables**	**Tertilesof Dietary Inflammatory Index**			
	**T1 (n=134)**	**T2 (n=135)**	**T3 (n=135)**	***P*** **trend** ^a^
MetS				
Crude	1.00	1.35 (0.82, 2.22)	0.98 (0.59, 1.63)	0.963
Model 1^b^	1.00	1.37 (0.81, 2.30)	1.03 (0.60, 1.78)	0.912
Model 2^c^	1.00	1.55 (0.89, 2.70)	1.15 (0.64, 2.07)	0.719
Model 3^d^	1.00	1.55 (0.84, 2.85)	0.92 (0.48, 1.76)	0.807
HDL (mg/dL)				
Crude	1.00	1.96 (1.12, 3.41)	2.29 (1.32, 3.97)	0.004
Model 1	1.00	2.05 (1.10, 3.81)	2.92 (1.54, 5.54)	0.001
Model 2	1.00	2.15(1.11, 4.15)	2.69 (1.35, 5.34)	0.005
Model 3	1.00	2.18 (1.11, 4.28)	2.71 (1.34, 5.47)	0.006
WC (cm)				
Crude	1.00	1.11 (0.68, 1.8)	1.19 (0.74, 1.94)	0.461
Model 1	1.00	1.00 (0.58, 1.73)	1.40 (0.80, 2.43)	0.215
Model 2	1.00	1.13 (0.65, 1.98)	1.39 (0.78, 2.47)	0.250
Model 3	1..00	1.00 (0.43, 2.31)	1.11 (0.45, 2.71)	0.816
FBS (mg/dL)				
Crude	1.00	1.55 (0.95, 2.52)	0.89 (0.54, 1.47)	0.672
Model 1	1.00	1.53 (0.90, 2.59)	0.88 (0.51, 1.53)	0.937
Model 2	1.00	1.58 (0.91, 2.73)	0.94 (0.52, 1.67)	0.833
Model 3	1.00	1.57 (0.90, 2.74)	0.87 (0.48, 1.57)	0.670
TG (mg/dL)				
Crude	1.00	0.89 (0.54, 1.47)	0.92 (0.56, 1.52)	0.764
Model 1	1.00	0.82 (0.47, 1.40)	0.78 (0.45, 1.37)	0.405
Model 2	1.00	0.84 (0.47, 1.49)	0.80 (0.44, 1.45)	0.471
Model 3	1.00	0.81 (0.45, 1.48)	0.71 (0.38, 1.32)	0.290
SBP (mm Hg)				
Crude	1.00	0.71 (0.42, 1.19)	0.57 (0.34, 0.95)	0.033
Model 1	1.00	0.90 (0.47, 1.75)	0.48 (0.41, 1.30)	0.230
Model 2	1.00	0.85 (0.43, 1.69)	0.46 (0.23, 1.94)	0.030
Model 3	1.00	0.87 (0.43, 1.73)	0.47 (0.22, 1.93)	0.029
DBP (mm Hg)				
Crude	1.00	0.67 (0.38, 1.18)	0.58 (0.31, 1.04)	0.07
Model 1	1.00	0.81 (0.40 1.61)	0.45 (0.22, 1.46)	0.51
Model 2	1.00	0.80 (0.39, 1.63)	0.46 (0.22, 1.12)	0.18
Model 3	1.00	0.80 (0.39, 1.64)	0.45 (0.21, 1.10)	0.12

Data are presented as odds ratio (95% CI). Abbreviations: WC, waist circumference; FBS, fasting blood glucose; TG, triglyceride; HDL, high-density lipoprotein; SBP, systolic blood pressure; DBP, diastolic blood pressure. ^a^Obtained by binary logistic regression. ^b^Model 1: adjusted for age, gender and energy intake. ^c^Model 2: additionally adjusted for marital status, physical activity, education status, smoking, economic status and supplementation. ^d^Model 3: further adjustment was made for BMI.

## Discussion

In this cross-sectional study, no significant association was found between adherence to a pro-inflammatory diet and odds of MetS. However, a significant positive association was found between DII score and reduced serum levels of HDL. No significant association was found in terms of other components of MetS. To the best of our knowledge; this is the first study that evaluated the association of DII scores with MetS and its components in the Middle East.

Prevalence of MetS is increased at an alarming rate in developed and developing countries.^27^ This syndrome is associated with other chronic diseases including diabetes, obesity, CVD and stroke which all decrease the quality of life and increase risk of morbidity and mortality.^26^ Inflammation is a potential risk factor for MetS. Recent studies have indicated that inflammatory potential of diet may have an important role in etiology of MetS like inflammation. However, findings in this regard are contradictory. In the present study, no significant association was found between the inflammatory potential of diet and odds of MetS. In line with our results, study of Wirth et al showed no significant association between DII and risk of MetS.^28^ Two other studies did not find any significant association between DII and MetS.^19,30^ However, in contrast to our study, a prospective study revealed a significant positive association between DII and MetS.^23^ In a cross-sectional study, adherence to a diet with high inflammatory potential was positively associated with odds of MetS in men and postmenopausal women.^31^ Another cross-sectional study on Iranian adult showed a significant association between DII and risk of MetS.^17^ However, Nikniaz et al study was conducted in a greater sample size than our study. Nikniaz et al study was done on 606 participants in East-Azarbaijan, Iran but our study was performed on 404 individuals from Tehran. The mean DII was 0.003 ± 1.61 that is less inflammatory compared with participants in another studies. The DII score of nearly 70% of the individual in this study was anti-inflammatory, this result indicates that the participant in present study had a healthy diet and because of this reason, no significant association was found between DII and MetS in this study. Different findings might be due to the differences in study designs, sample sizes, different food parameters used for DII calculation, different tools used for dietary intakes assessment and different criteria used for definition of MetS.

When we considered components of MetS, we observed that adherence to a diet with high inflammatory potential was positively associated with reduced levels of HDL-C. No other significant association was observed between DII and other components of MetS. Similar to our findings, a cross-sectional study conducted by Yosaee et al, indicated that higher scores of DII were associated with greater odds for having reduced concentrations of HDL-C.^5^ In another study, Pimenta et al reported an inverse association between the inflammatory potential of diet and serum levels of HDL-C.^18^ In opposite with our results, Naja et al found no significant association between DII and serum levels of HDL.^32^ Such non-significant association was also reported in two other studies.^5,33^ This discrepancy might be explained by different confounders which were adjusted in previous studies. For instance, in the study of Naja et al that found no significant association between DII and HDL concentrations, BMI as an important confounder was not controlled,^32^ but in our study, we adjusted for BMI and found a significant positive association between DII and reduced levels of HDL. Overall, further well-designed studies such as prospective design are warranted to reach a definite conclusion.

Although we found no significant association between DII and MetS in the current study, high inflammatory potential of diet may increase the risk of this syndrome by influencing serum concentrations of HDL. Diet with high inflammatory scores increases the production of inflammatory biomarkers which are inversely associated with HDL levels.^34^ Furthermore, the inflammatory potential of diet is positively associated with serum amyloid A levels that decrease the production of apolipoprotein A-I.^31^ High inflammatory potential of diet also decreases the levels of HDL through following mechanisms.

The strength of our study is that all data collection was performed by an expert dietitian through the valid and reliable questionnaires and cutoffs, thus all measurement was accurate. Moreover, sample size estimation is another strength of the study. However, the present study has some limitations that must be considered in the interpretation of the results. Due to the cross-sectional design of this study, causality cannot be established. Therefore, further studies with prospective design are needed. Moreover, in the present study, data on 30 food items were available for development of DII scores and 15 food items were missing that might influence our results. Missing parameters were as follow n-6 fatty acids, n-3 fatty acids, alcohol, ginger, saffron, turmeric, eugenol, flavones, flavonols, flavan-3-ol, flavonones, isoflavones, anthocyanidins, rosemary, and thyme/oregano. In addition, like other epidemiologic studies, the existence of measurement error and misclassification of study participants cannot be excluded in this study as well. However, a validated FFQ was used for assessment of dietary intakes in this study. Despite adjustment for several confounders in this study, the possible effects of residual confounders could not be controlled.

In conclusion, no significant association was found between DII scores and odds of MetS. However, a significant positive association was found between the inflammatory potential of diet and reduced concentrations of HDL-C. Other components of MetS were not significantly associated with the inflammatory potential of diet. Further studies, in particular those with prospective nature, are need to confirm the present findings.

## Competing interests

Authors declare that there is no conflict of interest.

## Ethical approval

This study was extracted from a PhD dissertation that was approved by School of Nutritional Sciences and Dietetics, Tehran University of Medical Sciences (Ethics No. 9423324003).
